# Ginsenoside Rg3 Ameliorates Stress of Broiler Chicks Induced by *Escherichia coli* Lipopolysaccharide

**DOI:** 10.3389/fvets.2022.878018

**Published:** 2022-04-08

**Authors:** Shicheng Bi, Yiwen Qu, Jianjian Shao, Jianrong Zhang, Weihao Li, Li Zhang, Jingxuan Ni, Liting Cao

**Affiliations:** ^1^Department of Traditional Chinese Veterinary Medicine, College of Veterinary Medicine, Southwest University, Chongqing, China; ^2^Immunology Research Center, Medical Research Institute, Southwest University, Chongqing, China

**Keywords:** ginsenoside, *Escherichia coli* lipopolysaccharide, broiler chicks, stress, inflammation

## Abstract

In broiler chicks*, Escherichia coli* lipopolysaccharide is a prominent cause for inflammatory damage and loss of immune homeostasis in broiler chicks. Ginsenosides have been shown to have anti-inflammatory and antioxidant effects. However, it has not been demonstrated that ginsenosides protect broiler chicks against stress induced by *Escherichia coli* lipopolysaccharide challenge. The aim of this is to investigate the protective effect of ginsenosides Rg1, Re, and Rg3 on *Escherichia coli* lipopolysaccharide-induced stress. Our results showed that Rg3 ameliorated growth inhibition and fever, as well as decreased the production of stress-related hormones in broilers with stress. The protective effect of Rg3 on the stressed chicks may be largely mediated by regulating inflammatory response and oxidative damage. Moreover, real-time quantitative-polymerase chain reaction (RT-qPCR) results demonstrated that Rg3 upregulated mRNA expression of *mTOR, HO-1*, and *SOD-1*. These results suggested that ginsenoside Rg3 and ginsenoside products contains Rg3 deserve further study for the control of immunological stress and inflammation in broiler chicks.

## Introduction

Gram-negative environmental pathogens, such as *Escherichia coli* (*E. coli*), are common pathogens that attack dairy cows, pigs, and chickens (1–3). These pathogenic microorganisms not only cause morbidity and mortality in animals but also lead to the loss of immune homeostasis, and trigger stress responses (4). Under stress, the anabolism of proteins and fats is weakened, while catabolism is promoted to meet the need for nutrients to synthesize immune effector molecules (5). In addition, clinical signs of stress such as depression and diarrhea induced growth inhibition in broilers, resulting in significant economic losses (6, 7). Thus, controlling the stress induced by *Escherichia coli* and other pathogenic microorganisms has become an urgent issue.

Lipopolysaccharide (LPS) is a main constituent of gram-negative bacterial cell walls and stimulates production of several proinflammatory cytokines such as interleukin-1β (IL-1β), interleukin-6 (IL-6), interleukin-8 (IL-8), and tumor necrosis factor alpha (TNF-α) via the toll-like receptor 4 (TLR4) signaling pathway (8). IL-6 may accelerate glycogen hydrolysis and glucose production while TNF-α may promote adipolysis and proteolysis, leading to reduced synthesis of protein and lipid (9, 10). Moreover, inflammatory cytokines could promote glucocorticoid secretion and reduce the secretion of growth hormone through the neuroendocrine system, resulting in decreased appetite and feed intake (11). In addition, LPS promoted secretion of several proinflammatory cytokines by increasing the phosphorylation of Jun N-terminal kinase (JNK), p38 mitogen-activated protein kinase (MAPK), nuclear factors kappa B (NF-κB) while decreasing the phosphorylation level of mammalian target of rapamycin (mTOR) (12). Because the overproduction proinflammatory cytokines can generate reactive oxygen species (ROS), the oxidative damage of multiple tissues should also be considered in the pathogenesis of LPS-induced stress (13). Therefore, inhibition of inflammatory response, oxidative damage as well as related pathways may be considered as important drug targets for the prevention of infection and immunological stress (14).

Ginsenosides Rg1, Re, and Rg3 are extracts made from the root of *Panax ginseng* C.A. Meyer, which has been used for thousands of years in traditional Chinese medicine (15). Previous studies have found that these agents exert anti-inflammatory and antioxidant effects via multiple pathways including TLR4/NF-κB (16–18). However, it has not been reported whether ginsenosides can protect broiler chicks against LPS-induced stress. In this study, we investigated the effects of ginsenosides Rg1, Rg3, and Re on stressed broiler chicks induced by *E. coli* LPS. Our results may reveal the potential role of ginsenosides in the prevention of immunological stress and inflammation in broiler chicks, as well as the underlying mechanism.

## Materials and Methods

### Reagents

**Figure 1:**
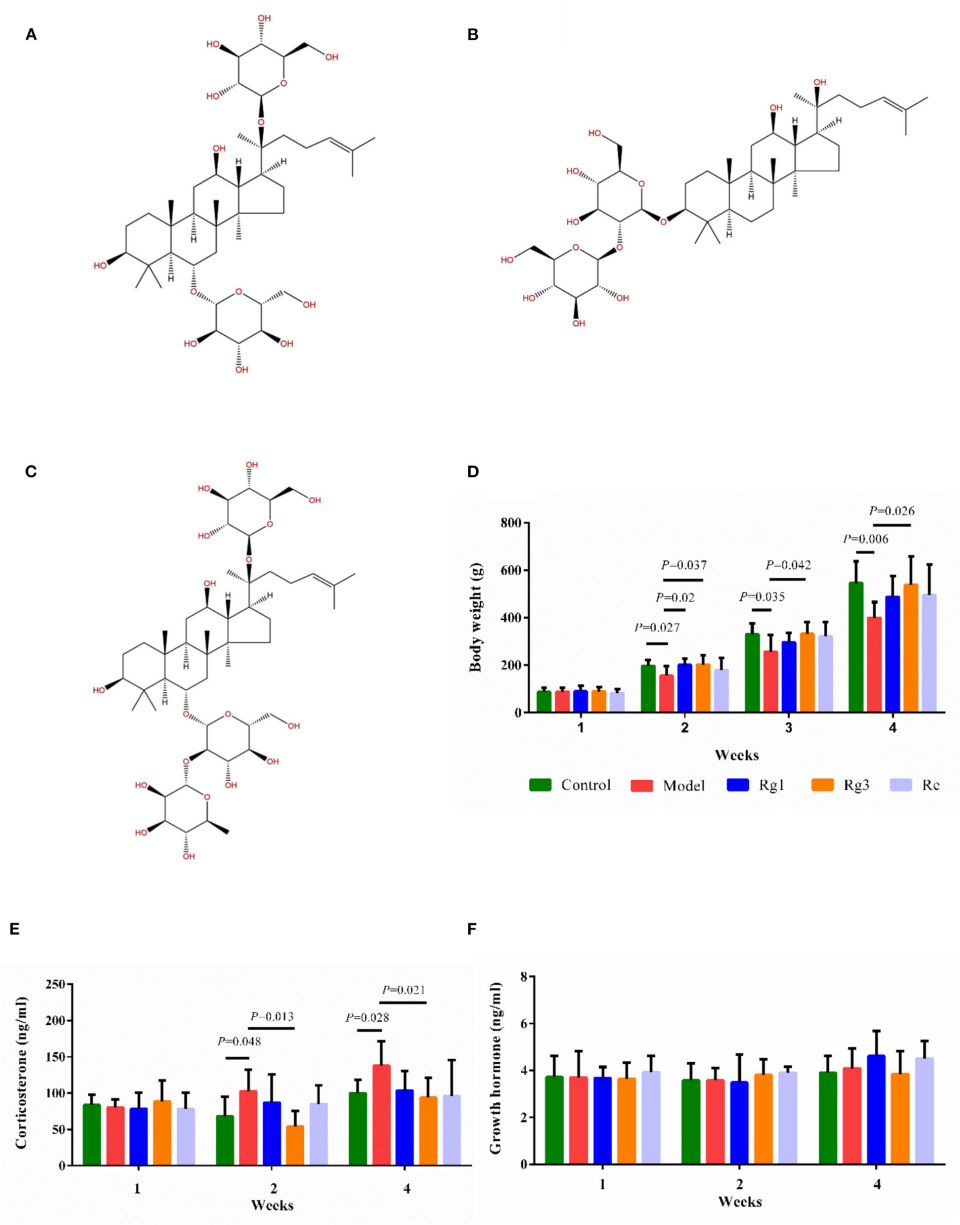
Effect of ginsenosides on growth and stress of broiler chicks. Chemical structure of ginsenoside Rg1 (C42H72O14; molecular weight, 801.024) **(A)**; Rg3 (C42H72O13; molecular weight, 785.025) **(B)**; Re (C48H82O18; molecular weight, 947.166) **(C)**. Determination of body weight **(D)**, serum corticosterone **(E)**, and serum growth hormone **(F)** by ELISA. Values are means ± standard error (SE) (*n* = 8).

Ginsenoside Rg1 (98.92%), Ginsenoside Rg3 (98.18%), and Ginsenoside Re (98%) were purchased from Chengdu Purify Biotechnology Co., Ltd. (Chengdu, China). The chemical structure of ginsenosides was shown in Figures 1A–C. Ginsenosides Rg1 (C_42_H_72_O_14_; molecular weight, 801.024), Re (C_48_H_82_O_18_; molecular weight, 947.166), and Rg3 (C_42_H_72_O_13_; molecular weight, 785.025) are extracted from the root of *Panax ginseng* C.A. Meyer. The majority of ginsenosides are dammarane-type saponins. According to the saponins, dammarane-type ginsenosides can be divided into two types, protopanaxadiol (PPD) and protopanaxatriol (PPT). Rg3 are PPD-type ginsenosides with sugar moieties attached to the β-OH at C-3 and/or C-20 in the aglycon PPD. Ginsenosides Re and Rg1 are PPT-type ginsenosides with sugar moieties linked to the α-OH at C-6 and/or β-OH at C-20 in the aglycon PPT. Although Rg1 and Re share a similar chemical structure with a dammarane skeleton, Re has an additional rhamnose at C-6 position.

Lipopolysaccharide from *E. coli* serotype O55:B5 was purchased from Sigma-Aldrich Chemical Co. (St. Louis, MO, USA). Detection kits for corticosterone (CORT), growth hormone (GH), and adrenocorticotropic hormone (ACTH) were purchased from Nanjing Jiancheng Bioengineering Institute (Nanjing, China). IL-6, IL-1β, TNF-α, and inducible nitric oxide synthase (iNOS) enzyme-linked immunosorbent assay (ELISA) kits were from Shanghai Lengdon Bioscienci Co., Ltd. (Shanghai, China). Detection kits for nitric oxide (NO), total superoxide dismutase (T-SOD), catalase (CAT), total antioxidant capacity (T-AOC), the contents of malondialdehyde (MDA), glutathione (GSH), glutathione peroxidase (GSH-PX), xanthine oxidase (XOD), and carbonyl were products of Nanjing Jiancheng Bioengineering Institute. All other chemicals were analytic grades.

### Experimental Design

Sichuan Lihua Poultry Co., Ltd. Provided one-day-old male Hongyu commecial broiler chickens, which were housed separately in three-layer cages. The indoor temperature was maintained at 32–34°C for a week, and then gradually decreased by 1°C every 2 day until a final temperature of 26°C was achieved. All broilers had free access to feed and water. The animal experiments were performed in accordance with the Animal Care and Use Committee of Southwest University (permit number IACUC-20200701-01). All experimental animals were euthanized at the end of the experiment.

In experiment 1, 40 broiler chicks were randomly divided into 5 groups, each consisting of 8 birds (Table 1). Chicks were received intraperitoneally administered saline, 1 mg Rg1/kg body weight (1 mg/kg Rg1), 1 mg/kg Re, or 1 mg/kg Rg3 2 h before LPS challenge. The chicks were then given intraperitoneal injection of 250 μg/kg LPS at the ages of 12, 14, 33, and 35 days to induce immunological stress (19). Control group was injected with an equivalent amount of sterile saline. Body weight was measured every week. Blood samples were collected from 8 chickens at the age of 1, 2, and 4 weeks for determination of CORT and GH.

**Table 1:** Experimental design (experiment 1).

**Group**	***n***	**LPS**	**Drug**
Control	8	Saline	Saline
Model	8	250 μg/kg	Saline
Rg1	8	250 μg/kg	1 mg/kg Rg1
Rg3	8	250 μg/kg	1 mg/kg Rg3
Re	8	250 μg/kg	1 mg/kg Re

In experiment 2, one hundred and sixty broiler chicks were randomly allocated to 4 treatments. Each treatment contained 4 replicates of 10 broilers per replicate (Table 2). Chickens in Rg3-treated groups were received intraperitoneally administered 1 mg/kg Rg3 2 h before or after LPS challenge. Control group was injected with an equivalent amount of sterile saline. Then the broilers were intraperitoneally injection of 250 μg/kg LPS at the age of 12, 14, 33, and 35 days to induce immunological stress. Birds were group weighed by cage at the age of 7, 14, 21, 28, and 35 days. Feed intake was monitored by cage at the age of 7, 14, 21, 28, and 35 days. Average daily weight gain (DWG), daily feed intake (DFI), and feed conversion ratio (FCR) were calculated for each period and for the overall experiment. At the age of 12 days, 16 chickens were randomly selected from each group. A digital thermometer for livestock (LY-302C, Lvyuan Hengtai, Co., Ltd., China) was used to measure body temperature by inserting it into the cloaca. The results were recorded every 4 h and monitored for 24 h continuously. Blood samples were collected using wing vein puncture at 1, 2, and 4 weeks and the serum was obtained for detection of hormone content, inflammatory mediators and antioxidant parameters. Total RNA was extracted from liver for real-time quantitative polymerase chain reaction (RT-qPCR analysis).

**Table 2:** Experimental design (experiment 2).

**Group**	***n***	**LPS**	**Drug**
Control	40	Saline	Saline
Model	40	250 μg/kg	Saline
Rg3-Pre	40	250 μg/kg	1 mg/kg Rg3
Rg3-Post	40	250 μg/kg	1 mg/kg Rg3

### Determination of CORT, GH, and ACTH

About 4 mL of blood was centrifuged at 3,000 g for 10 min to get serum. The concentrations of serum CORT (H205), GH (H091-1-2), and ACTH (H097-1-2) were determined by using ELISA kits (Jiancheng Bioengineering Institute, Nanjing, China) according to the manufacturer's instructions.

### Measurement of Inflammatory Responses

The contents of IL-6, IL-1β, TNF-α, NO, and iNOS in the serum samples were measured by ELISA using chicken-specific quantification kits (Lengdon Bioscienci Co., Ltd., Shanghai, China).

### Assay of Antioxidant Enzymes

The enzyme activities of T-SOD (A001-1-2), CAT (A007-1-1), T-AOC (A015-2-1), GSH-Px (A005-1-2), XOD (A002-1-1), and the contents of MDA (A003-1-2), GSH (A006-2-1), carbonyl (A087-1-2) in the serum were determined spectrophotometrically with the commercial kits (Jiancheng Bioengineering Institute, Nanjing, China) according to the instructions of the manufacturer.

### RNA Extraction and RT-qPCR

Total RNA was extracted from the liver using TRIzol reagent (Takara, Shiga, Japan) following the manufacturer's guidelines. PrimeScript™RT Master Mix (Takara, Dalian, China) was utilized to convert RNA into cDNA on a T100™ thermal cycler (Bio Rad, Hercules, CA). The Chicken β-actin was served as the internal control gene. RT-qPCR with SYBR®Premix Ex Taq™ II (Tli RNaseH Plus) (Takara, Dalian, China) on selected genes was performed on a Multiple Real-Time PCR System (Applied Biosystems, Carlsbad, CA). A relative quantitative method (2^−ΔΔCT^) was employed to evaluate the quantitative variation (20). The primer sequences were list in Table 3. The final primer concentration was 10 pmol/μl of each primer.

**Table 3:** Sequences of primers for RT-qPCR.

**Gene**	**F/R**	**Primer sequence (5′-3′)**	**Product size (bp)**	**References**
*β-actin*	F	GCCAACAGAGAGAAGATGACAC	118	(21)
	R	GTAACACCATCACCAGAGTCCA		
*Akt*	F	AGGAGGAAGAGATGATGGAT	290	(22)
	R	GAATGGATGCCGTGAGTT		
*MyD88*	F	CCTGGCTGTGCCTTCGGA	198	(21)
	R	TCACCAAGTGCTGGATGCTA		
*NF-κB p65*	F	CAGCCCATCTATGACAACCG	151	(21)
	R	CAGCCCAGAAACGAACCTC		
*SOD-1*	F	CCGGCTTGTCTGATGGAGAT	125	(13)
	R	TGCATCTTTTGGTCCACCGT		
*mTOR*	F	AACCACTGCTCGCCACAATGC	120	(23)
	R	CATAGGATCGCCACACGGATTAGC		
*PI3K*	F	AGAGCGTGTGCCCTTTGTCTTAAC	135	(23)
	R	TGCTGCCGAATTGCTAGATATGCC		
*HO-1*	F	GGTCCCGAATGAATGCCCTTG	138	(24)
	R	ACCGTTCTCCTGGCTCTTGG		

### Statistics Analysis

The one-way analysis of variance with Duncan's multiple range test was employed using SPSS software (version 20.0, SPSS Inc., Chicago, IL). Data was expressed as mean ± standard error (*SE*). Differences between means at *p* < 0.05 or *p* < 0.01 were statistically significant.

## Results

### Body Weight and Growth Performance

In experiment 1, the effect of each treatment on body weight was shown in Figure 1D. The LPS challenge significantly decreased body weight of broilers at the age of 2 (*p* < 0.05), 3 (*p* < 0.05), and 4 (*p* < 0.01) weeks. However, administration of Rg1 (2 weeks of age, *p* < 0.05) or Rg3 (2, 3, and 4 weeks of age, *p* < 0.05) attenuated LPS-induced growth inhibition.

In experiment 2, the effect of Rg3 on growth performance of broiler chicks was depicted in Table 4. The chicks with stress (Model) had lower DWG (3 weeks, *p* < 0.05; 4 weeks, *p* > 0.05; 5 weeks, *p* > 0.05) and DFI (3 weeks, *p* < 0.05; 4 weeks, *p* > 0.05; 5 weeks, *p* > 0.05) than the Controls. However, chicks pre-treated or post-treated with Rg3 had higher DFI at 3 weeks (*p* < 0.05), 4 weeks (*p* > 0.05), and 5 weeks (*p* > 0.05) than the Models. Furthermore, pre-treatment with Rg3 numerically increased the DWG of stressed chicks during any periods. There were no significant differences in FCR of broilers between treatments (*p*> 0.05).

**Table 4:** Growth performance (experiment 2).

**Item**	**Group**			
	**Control**	**Model**	**Rg3-Pre**	**Rg3-Post**
**DWG** **(g/bird per day)**				
2 weeks	13.21 ± 0.42	13.34 ± 0.11	12.36 ± 0.55	13.09 ± 0.42
3 weeks	20.31 ± 0.23^a^	18.65 ± 0.24^b^	19.65 ± 1.17^ab^	19.30 ± 0.75^ab^
4 weeks	28.92 ± 0.75	28.28 ± 0.91	28.96 ± 1.45	28.27 ± 2.28
5 weeks	29.44 ± 1.61	26.85 ± 1.46	27.96 ± 2.06	30.13 ± 1.75
**DFI** **(g/bird per day)**				
2 weeks	25.70 ± 0.54	26.26 ± 0.45	25.65 ± 0.31	25.62 ± 0.40
3 weeks	43.18 ± 0.44^a^	36.45 ± 0.04^d^	40.58 ± 0.37^b^	40.07 ± 0.56^bc^
4 weeks	55.84 ± 1.48	52.79 ± 2.26	54.95 ± 0.59	53.53 ± 1.46
5 weeks	78.86 ± 2.30	71.26 ± 2.46	75.05 ± 2.18	74.60 ± 1.57
**FCR**				
2 weeks	1.95 ± 0.06	1.97 ± 0.03	2.09 ± 0.11	1.96 ± 0.07
3 weeks	2.13 ± 0.04	2.06 ± 0.08	2.07 ± 0.08	2.14 ± 0.09
4 weeks	1.98 ± 0.09	1.92 ± 0.05	1.91 ± 0.08	1.94 ± 0.15
5 weeks	2.70 ± 0.19	2.82 ± 0.19	2.75 ± 0.26	2.50 ± 0.18

*DWG, average daily weight gain; DFI, daily feed intake; FCR, feed conversion ratio. Values were expressed as means ± SE. The different superscript letters represent statistically different*.

### Serum CORT and GH

In experiment 1, LPS challenge distinctly increased serum CORT (2 weeks, *p* < 0.05; 4 weeks, *p* < 0.05) content in broilers compared to the Control (Figure 1E). However, pre-treatment with Rg3 significantly decreased serum CORT (2 weeks, *p* < 0.05; 4 weeks, *p* < 0.05) content of broilers after LPS challenge. In addition, there was no significant difference in serum GH of broilers among the different groups (Figure 1F).

### Body Temperature

**Figure 2:**
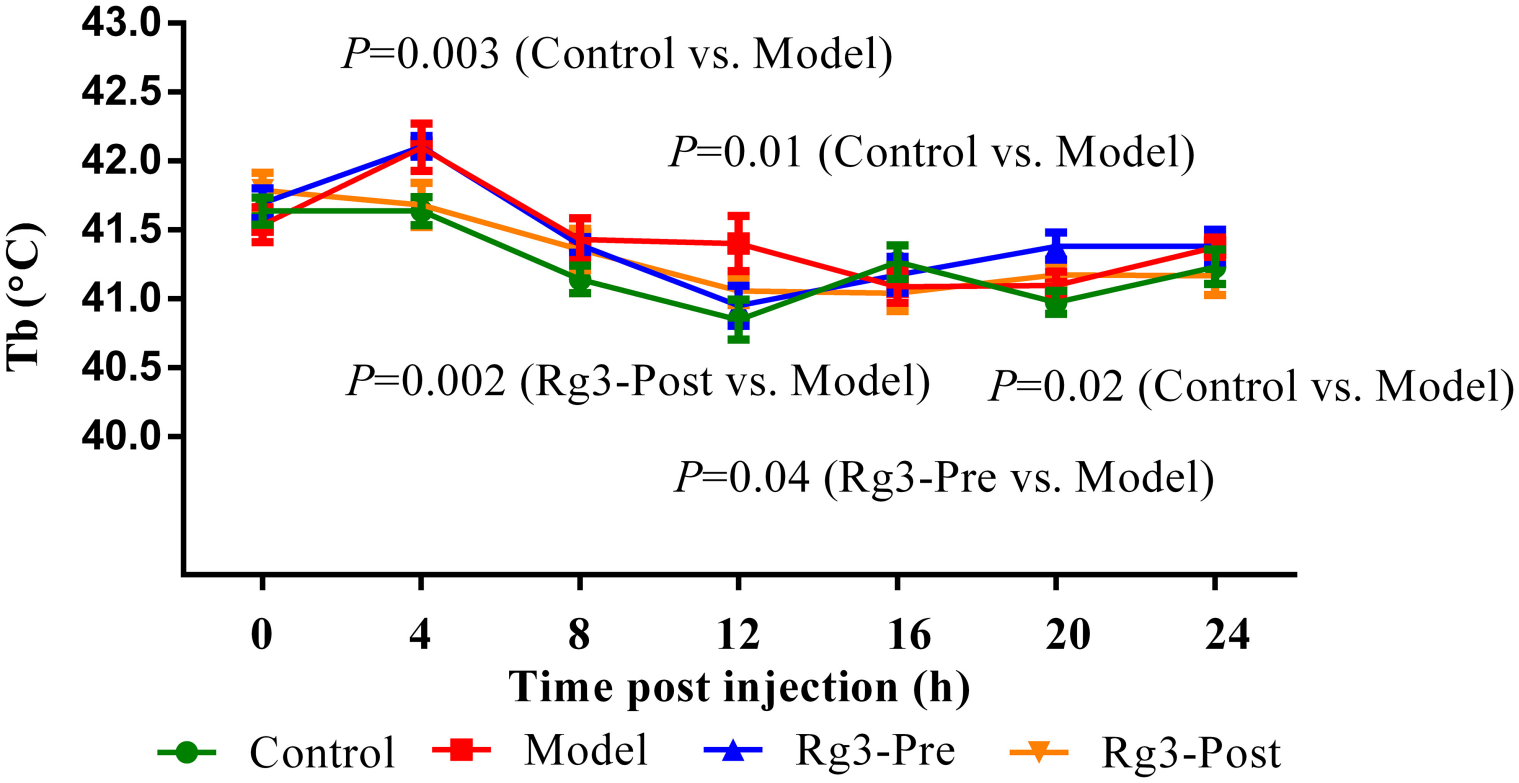
Continuous body temperature (Tb) record in broilers. Continuous monitoring of body temperature post-lipopolysaccharide injection. Values are represented as means ± SE (*n* = 16).

The body temperature of broilers at 2 weeks was recorded. As depicted in Figure 2, there was no significant difference (*p*> 0.05) in body temperature among the four groups of broilers before LPS challenge. After the LPS injection, the body temperature increased rapidly, peaked at 4 h (*p* < 0.01), and returned to baseline levels at 16 h. However, the body temperature of broilers pre-treated with Rg3 decreased at 12 h, 4 h earlier than broilers injected with LPS only (*p* < 0.05). In addition, the body temperature of stressed broilers post-treated with Rg3 was lower than broilers that only injected with LPS at 4 h (*p* < 0.01), 8 h (*p* > 0.05), 12 h (*p* > 0.05), 16 h (*p*> 0.05), and 24 h (*p* > 0.05). The body temperature of stressed broilers pre-treated with Rg3 was numerically lower than broilers that only injected with LPS at different timepoint (*p* > 0.05).

### ACTH and Inflammatory Responses in Serum of Broilers

**Figure 3:**
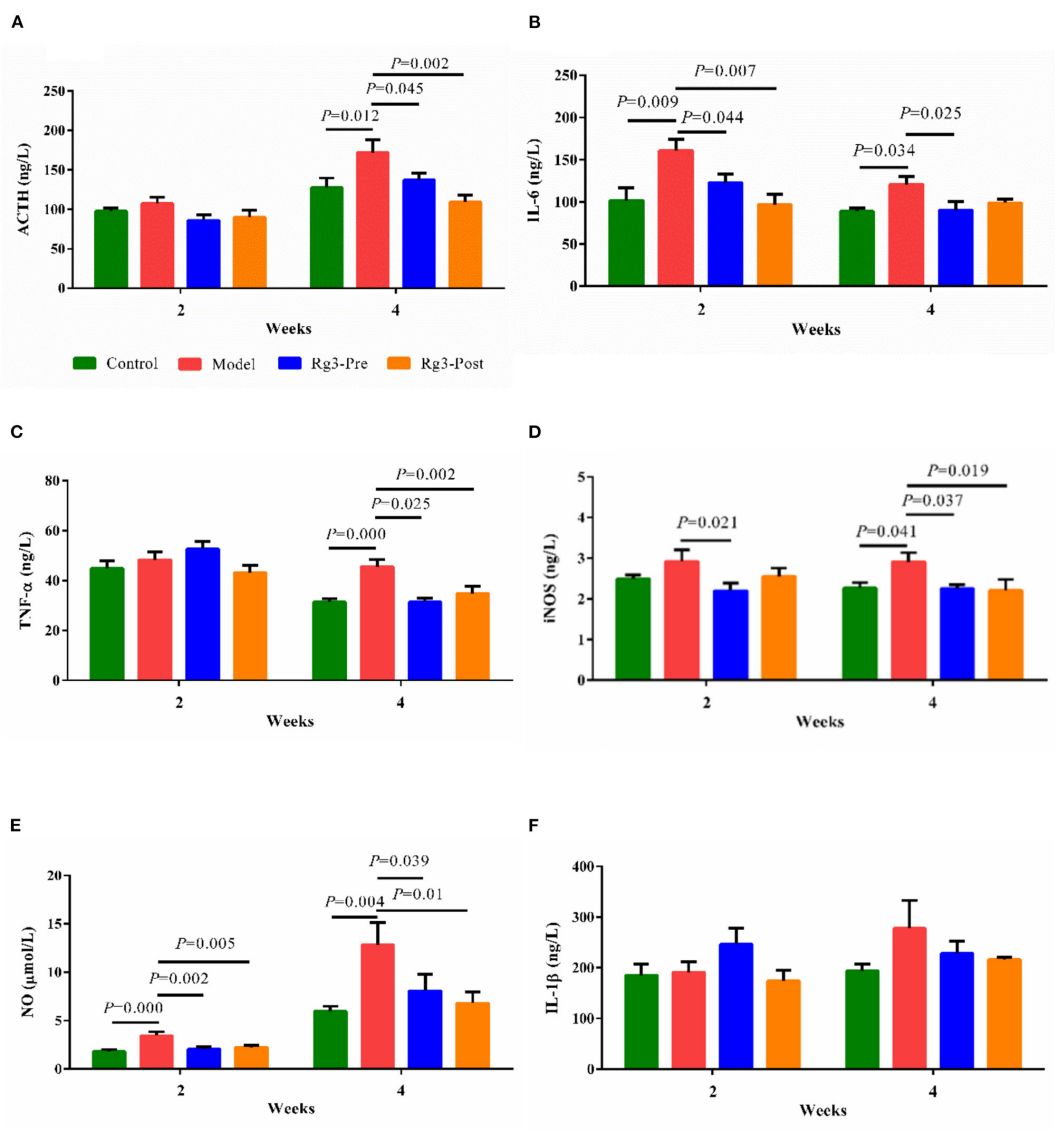
Effect of ginsenoside Rg3 on adrenocorticotropic hormone (ACTH) and inflammatory responses in serum of broilers with stress. Determination of serum ACTH **(A)**, interleukin-6 (IL-6) **(B)**, tumor necrosis factor α (TNF-α) **(C)**, inducible nitric oxide synthase (iNOS) **(D)**, nitric oxide (NO) **(E)**, and interleukin-1β (IL-1β) **(F)** by ELISA. The values are represented as means ± SE (*n* = 10).

In experiment 2, LPS challenge increased serum ACTH (2 weeks, *p* > 0.05; 4 weeks, *p* < 0.05) content, when compared with the Control. However, pre-treatment with Rg3 decreased serum ACTH (2 weeks, *p* > 0.05; 4 weeks, *p* < 0.05) content of broilers after LPS challenge. Post-treated with Rg3 also decreased serum ACTH (2 weeks, *p* > 0.05; 4 weeks, *p* < 0.01) content after LPS challenge (Figure 3A).

The effect of Rg3 on inflammatory responses of stressed broilers was shown in Figures 3B–F. Results showed that the LPS challenge promoted the production of serum IL-6 (2 wk, *p* < 0.01; 4 wk, *p* < 0.05), TNF-α (2 weeks, *p* > 0.05; 4 weeks, *p* < 0.01), iNOS (2 weeks, *p* > 0.05; 4 weeks, *p* < 0.05), and NO (2 week, *p* < 0.01; 4 weeks, *p* < 0.01). However, pre-treatment of Rg3 inhibited the increased IL-6 (2 weeks, *p* < 0.05; 4 weeks, *p* < 0.05), TNF-α (2 weeks, *p* > 0.05; 4 weeks, *p* < 0.05), iNOS (2 weeks, *p* < 0.05; 4 weeks, *p* < 0.05), and NO (2 weeks, *p* < 0.01; 4 weeks, *p* < 0.05). In addition, post-treatment of Rg3 also reversed the increase of IL-6 (2 weeks, *p* < 0.01; 4 weeks, *p* > 0.05), TNF-α (2 weeks, *p* > 0.05; 4 weeks, *p* < 0.01), iNOS (2 weeks, *p* > 0.05; 4 weeks, *p* < 0.05), and NO (2 weeks, *p* < 0.01; 4 weeks, *p* < 0.05). There was no significant difference in serum IL-1β of broilers among the different groups.

### Effect of Rg3 on Serum Oxidation and Antioxidant Indices

**Figure 4:**
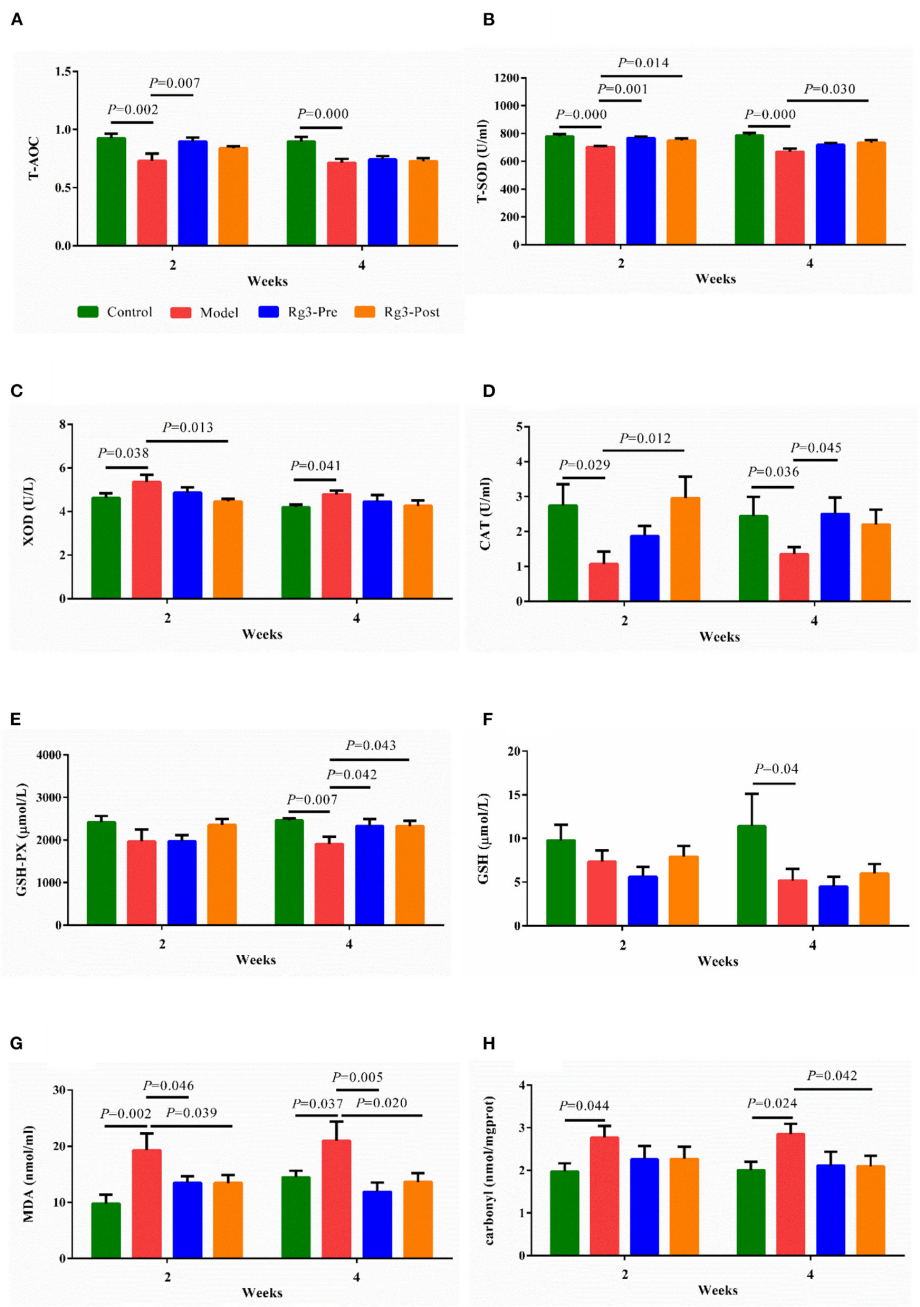
Effect of ginsenoside Rg3 on enzymatic antioxidants and non-enzymatic antioxidants in broilers with stress. Detection for serum total antioxidant capacity (T-AOC) **(A)**, total superoxide dismutase (T-SOD) **(B)**, xanthine oxidase (XOD) **(C)**, catalase (CAT) **(D)**, glutathione peroxidase (GSH-PX) **(E)**, glutathione (GSH) **(F)**, malondialdehyde (MDA), **(G)** and carbonyl **(H)**. Values are means ± SE (*n* = 10).

As shown in Figures 4A–E, injection of LPS decreased the activities of T-AOC (2 weeks, *p* < 0.01; 4 weeks, *p* < 0.01), T-SOD (2 weeks, *p* < 0.01; 4 weeks, *p* < 0.01), CAT (2 weeks, *p* < 0.05; 4 weeks, *p* < 0.05), GSH-PX (2 weeks, *p* > 0.05; 4 weeks, *p* < 0.01), and increased the activity of XOD (2 weeks, *p* < 0.05; 4 weeks, *p* < 0.05) compared to the Control. However, pre-treatment of Rg3 inhibited decreases in activities of T-AOC (2 weeks, *p* < 0.01; 4 weeks, *p* > 0.05), T-SOD (2 weeks, *p* < 0.01; 4 weeks, *p* > 0.05), CAT (2 weeks, *p* > 0.05; 4 weeks, *p* < 0.05), and GSH-PX (2 weeks, *p* > 0.05; 4 weeks, *p* < 0.05) in broilers challenged with LPS. Meanwhile, post-treated with Rg3 inhibited decreases in activities of T-SOD (2 weeks, *p* < 0.05; 4 weeks, *p* < 0.05), CAT (2 weeks, *p* < 0.05; 4 weeks, *p* > 0.05), GSH-PX (2 weeks, *p* > 0.05; 4 weeks, *p* < 0.05), and decreased the activities of XOD (2 weeks, *p* < 0.05; 4 week, *p* > 0.05) in chicks challenged with LPS.

As illustrated in Figures 4F–H, LPS challenge reduced the content of serum GSH (2 weeks, *p* > 0.05; 4 weeks, *p* < 0.05), and increased the levels of MDA (2 weeks, *p* < 0.01; 4 weeks, *p* < 0.05), and Carbonyl (2 weeks, *p* < 0.05; 4 weeks, *p* < 0.05), when compared with the Control. However, pre-treatment of Rg3 inhibited elevation of MDA (2 weeks, *p* < 0.05; 4 weeks, *p* < 0.01) in serum of chickens challenged with LPS. In addition, post-treated with Rg3 reversed increase of MDA (2 weeks, *p* < 0.05; 4 weeks, *p* < 0.05), and carbonyl (2 weeks, *p*> 0.05; 4 weeks, *p* < 0.05) induced by LPS challenge.

### Relative Expression of mRNA

**Figure 5:**
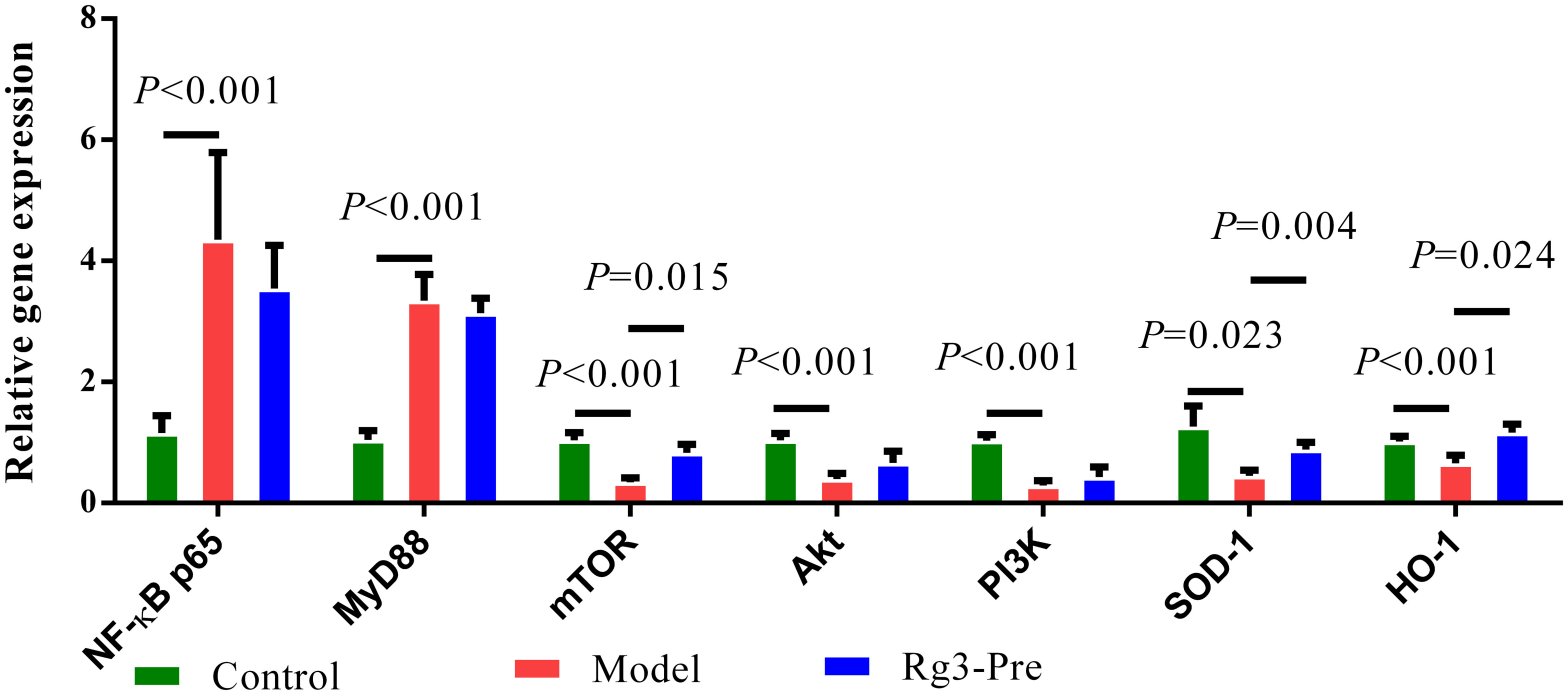
Relative mRNA expression in the liver. Total RNA was extracted from the liver. The Chicken β-actin was served as the internal control gene. Expression of nuclear factors kappa B (NF-κB) p65, myeloid differential protein-88 (MyD88), mammalian target of rapamycin (mTOR), AKT serine-threonine kinase 1 (Akt), phosphoinositol 3 kinase (PI3K), and SOD-1 mRNA were assessed by real-time quantitative PCR. A relative quantitative method (2^−ΔΔCT^) was employed to evaluate the quantitative variation. Data are represented as means ± SE (*n* = 6).

As shown in Figure 5, significantly elevated mRNA expression of *NF-*κ*B p65* (*p* < 0.01), *MyD88* (*p* < 0.01), and significantly decreased mRNA expression of *mTOR* (*p* < 0.01), *Akt* (*p* < 0.01), *PI3K* (*p* < 0.01), *SOD-1* (*p* < 0.05), and *HO-1* (*p* < 0.01) were detected in the liver of broilers with stress (Model), when compared with the Control. However, pretreatment of Rg3 significantly increased mRNA expression of *mTOR* (*p* < 0.05), *SOD-1* (*p* < 0.01), and *HO-1* (*p* < 0.05). There was no significant difference in mRNA expression of *NF-*κ*B p65, MyD88, Akt*, and *PI3K* between the Model and Rg3-Pre-group.

## Discussion

Young broilers are more susceptible to pathogens than adults because of their immature immune system and high demand for nutrients (25–27). Thus, nutritional interventions were commonly used to maintain growth performance and alleviate the harmful consequences of bacterial LPS challenge (13, 28, 29). Nevertheless, the relief of fever, depression, and diarrhea plays a critical role for animals to recover from immunological stress (19, 30, 31). Plant extracts such as ginseng stem and leaf saponin (GSLS) and astragalus polysaccharide are found effective to regulate inflammation and oxidative stress (19, 32). In 2017, the Agricultural Ministry of China issued a certificate approving the product made from GSLS to be used in poultry (2017–20). It is interesting whether ginsenosides Rg1, Rg3, and Re in GSLS could protect broilers chicks from stress induced by *E. coli* LPS. The results showed that Rg3 could improve the growth inhibition, shorten the duration of fever and the maximum body temperature, and decrease the serum CORT and ACTH in stressed broilers. Ultimately, Rg3 reversed the growth inhibition and attenuated the stress. In the future, detection of Rg3 content in ginsenosides can be used as one of approaches to control quantity of ginsenosides products.

Although remarkedly decreased body weight gain was observed in broilers challenged with LPS, there was no significant difference in serum growth hormone between Model and Control groups. Generally, the growth inhibition of broilers with immunological stress might be attributed to the activation of inflammatory responses (13, 31). Increased inflammatory cytokines alter metabolic process in cells, depleting nutrition of the immune system, and inhibiting the growth and development of animals (9, 10). Therefore, regulation of pro-inflammatory cytokines and inflammatory mediators is wanted to prevent LPS-induced stress (27, 33). In this study, Rg3 inhibited the production of IL-6, TNF-α, NO, and iNOS in a bird model, which gives us an enlightenment to use Rg3 and other ginsenosides to control inflammation-related diseases in the poultry industry. Our data was consistent with previous results. Yoon et al. (34) found that two optical isomers of Rg3 could suppress NO generation and iNOS expression in LPS-stimulated peritoneal macrophages and inhibit IL-1β production induced by inflammasome activation. Shi et al. (35) reported the inhibiting effect of Rg3 on inflammation evidenced by decreased IL-1 secretion and blocked activation of the caspase-1 and the NLRP3 inflammasome in human and mouse macrophages.

The production of proinflammatory cytokines (TNF-α, IL-1β, and IL-6) and inducible proinflammatory enzymes such as iNOS-released free radicals and induced oxidative stress, causing cell apoptosis and tissue damage (36). Interestingly, we observed the reduced content of MDA and Carbonyl, and the increased activity of T-AOC, T-SOD, GSH-Px, and CAT in the serum of LPS-challenged broilers pre-treated or treated with Rg3. Therefore, we inferred that Rg3 protected stressed broilers also by attenuating the oxidative damage. Numerous studies have confirmed the ability of Rg3 to protect against oxidative stress *in vitro* and *in vivo* (18, 37).

Cytokines such as IL-1β, IL-6, and TNF-α have been implicated as mediators to induce fever, and subsequently increase basal metabolite rate and decrease growth performance. Thus, inhibition of cytokines was generally thought as a strategy to attenuate fever and stress (27, 33). In the current study, reduction of maximum body temperature and the duration of fever were associated with decreased production of cytokines in Rg3-treated groups, which is consistent with previous reports. Thus, we presumed Rg3 might regulate the body temperature by suppression of neuroinflammation (38).

The mTOR is a conserved Ser/Thr kinase and is most well-known for mediating the signaling of nutrition, especially branched chain amino acids and lipid metabolism (39, 40). In addition, plant extracts have been reported to regulate inflammation through multiple signal pathways including mTOR. Resveratrol protected the myocardium in sepsis by activating the PI3K/AKT/mTOR pathway and inhibiting the NF-κB signaling pathway (41). Scoparone ameliorated hepatic inflammation of mice with nonalcoholic steatohepatitis by regulating the ROS/P38/Nrf2 axis and PI3K/AKT/mTOR pathway in macrophages (42). In the current study, RT-qPCR assays showed that mRNA expression of *mTOR, SOD-1*, and *HO-1* was increased in the stress of broilers with the administration of ginsenoside Rg3. These results suggested that the protective effect of Rg3 on stress might be related to upregulation of mTOR and antioxidant enzymes. Activation of mTOR or HO-1 has been shown to suppress NF-κB, thus eventually inhibit the inflammation (41, 43). We observed inhibited inflammatory responses in chickens treated with Rg3, however, the motion that whether Rg3 inhibited inflammatory responses by suppressing activation of NF-κB needs to further demonstrate.

In conclusion, ginsenoside Rg3 could reduced the stress of broiler chicks induced by *E. coli* LPS. The effect of Rg3 on stress may be related to the inhibition of inflammatory response, protection of oxidative damage, and upregulation of *mTOR, HO-1, SOD-1*. In addition, plant extracts contain Rg3 deserve further study for the control of immunological stress and inflammation in broiler chicks.

## Data Availability Statement

The original contributions presented in the study are included in the article/supplementary material, further inquiries can be directed to the corresponding author/s.

## Ethics Statement

The animal study was reviewed and approved by Southwest University Committee on Animal Care and Use.

## Author Contributions

SB and LC conceived and designed the experiments. SB, YQ, JS, JZ, and WL performed the experiments. YQ, JS, LZ, and JN analyzed the data. SB wrote the paper. All authors read and approved the final manuscript.

## Funding

This work was supported by the National Natural Science Foundation of China (32002325), Chongqing Research Program of Basic and Frontier Technology (cstc2020jcy-jmsxmX0418), and Fundamental Research Funds for the Central Universities (SWU119054).

## Conflict of Interest

The authors declare that the research was conducted in the absence of any commercial or financial relationships that could be construed as a potential conflict of interest.

## Publisher's Note

All claims expressed in this article are solely those of the authors and do not necessarily represent those of their affiliated organizations, or those of the publisher, the editors and the reviewers. Any product that may be evaluated in this article, or claim that may be made by its manufacturer, is not guaranteed or endorsed by the publisher.
